# Supplementary material for: Prioritization of candidate causal genes for asthma in susceptibility loci derived from UK Biobank
Source: Commun Biol. 2021 Jun 8;4:700. doi: 10.1038/s42003-021-02227-6 (PMC8187656; doi:10.1038/s42003-021-02227-6)
Supplement: Supplementary file 2 — Supplementary information [file 42003_2021_2227_MOESM2_ESM.pdf]

Supplementary information

Prioritization of candidate causal genes for asthma in susceptibility loci derived from UK Biobank

Kim Valette[1], Zhonglin Li[1], Valentin Bon-Baret[1], Arnaud Chignon[1], Jean-Christophe Bérubé[1], Aida Eslami[1], Jennifer Lamothe[1], Nathalie Gaudreault[1], Philippe Joubert[1], Ma'en Obeidat[2], Maarten van den Berge[3], Wim Timens[4], Don D. Sin[2], David Nickle[5], Ke Hao[6], Catherine Labbé[1], Krystelle Godbout[1], Andréanne Côté[1], Michel Laviolette[1], Louis-Philippe Boulet[1], Patrick Mathieu[1], Sébastien Thériault[1,7], Yohan Bossé[1,8]

Supplementary Table 1. Investigational or approved asthma drugs acting on identified gene targets

**Target genes**	**Drugs**	**Action type**
CCR4	MOGAMULIZUMAB	Cross-linking agent
CSF2	LENZILUMAB	Inhibitor
IL13	ANRUKINZUMAB<br>LEBRIKIZUMAB<br>DECTREKUMAB<br>TRALOKINUMAB	Inhibitor<br>Inhibitor<br>Inhibitor<br>Inhibitor
IL1R1	ANAKINRA	Antagonist
IL23A	RISANKIZUMAB	Inhibitor
IL2RA	DACLIZUMAB	Inhibitor
IL4	PASCOLIZUMAB	Inhibitor
IL4R	**DUPILUMAB**	Antagonist
IL5	**MEPOLIZUMAB**<br>**RESLIZUMAB**	Inhibitor<br>Inhibitor
IL6	CLAZAKIZUMAB<br>SIRUKUMAB	Inhibitor<br>Inhibitor
TNF	ADALIMUMAB<br>ETANERCEPT<br>GOLIMUMAB	Inhibitor<br>Inhibitor<br>Inhibitor
TNFSF4	OXELUMAB	Inhibitor
TSLP	TEZEPELUMAB	Inhibitor

In bold are drugs that have demonstrated clinical efficacy in phase 3 clinical trials.

Supplementary Table 2. Druggable target genes consistently identified across methods

Genes	Asthma score*	Z TWAS	P value	Drug	Interaction	Indication
CAMK4	0.165	5.934	2.94e-09	CHEMBL261720	NA	Experimental
				ESTRADIOL BENZOAT	NA	Oestrogenic hormonal therapy
				GEMCITABINE	NA	Antineoplastic agent (solid cancers)
HSPA4	0.088	5.999	1.98e-09	LITHIUM	NA	Antipsychotic (mania, depression)
				PUROMYCIN	NA	For cell culture (microbiology), no clinical research or application
				ARSENIC TRIOXIDE	NA	Antineoplastic agent (oncohematology)
				BORTEZOMIB	NA	Antineoplastic agent (oncohematology)
				CELECOXIB	NA	Anti-inflammatory and anti-rheumatic drug, non-steroids<br>Investigational as antineoplastic agent
				CHLORPROMAZINE	NA	Neuroleptic antipsychotic, sedative antihistamine
				CYTARABINE	NA	Antineoplastic agent (oncohematology)
				DEFEROXAMINE	NA	Iron chelating agent
				6-DIAZO-5-OXO-L-NORLEUCINE	NA	Experimental as antineopslastic agent
				ENALAPRIL	NA	Angiotensin converting enzyme inhibitor antihypertensive
				EPOIETIN ALFA	NA	Antianemic agent
				FLUTICASONE PROPIONATE	NA	Inhaled corticosteroid used in local treatment of asthma and COPD
				GOSSYPOL	NA	Experimental as contraceptive and antineoplastic agent
				HALOPERIDOL	NA	Neuroleptic antipsychotic
				HEPARIN	NA	Antithrombotic agent
				HYDRALAZINE	NA	Vasodilatator agent, antihypertensive
				IFOSFAMIDE	NA	Antineoplastic agent
				KETANSERIN	NA	Serotonin antagonist, antihypertensive agent
				NIFEDIPINE	NA	Calcium channel blockers (antihypertensive, vasodilatator)
				NIMESULIDE	NA	Non steroid anti-inflammatory
				PHENYLEPHRINE	NA	Adrenergic agent, vasoconstrictor (hypotension treatment)
				PHOTOPHRIN	NA	Photosensitizer for palliative photodynamic therapy of obstructive cancer
				MIDOSTAURIN	NA	Antineoplastic agent (oncohematology)
				PERILLYL ALCOHOL	NA	Experimental as antineoplastic agent
				RANITIDINE	NA	H2 receptor antagonist drug for ulcer or gastro-oesophageal reflux disease
				SODIUM CHLORIDE	NA	Mineral supplement fluid (hydratation)
				SODIUM SALICYLATE	NA	Analgesic, antipyretic drug
				THIABENDAZOLE	NA	Experimental as antineoplastic agent
				UREA	NA	Keratolytic agent(onychomycosis)
				VERAPAMIL	NA	Calcium channel blocker (antihypertensive, antiarythmic)
				ASCORBATE	NA	Water-soluble C vitamin (deficiency, infectious disease)
				WORTMANNIN	NA	Experimental as antineoplastic agent
				ISOPROTERENOL	NA	Beta-adrenergic stimulant (cardiac stimulant, bronchodilatator)
				ZALCITABINE	NA	Antiretroviral agent (HIV)
IL4R	1.000	-5.818	5.94e-09	CINTREDEKIN BESUDOTOX	NA	Investigational as anti-neoplastic agent (brain cancer)
				SILYBIN B	agonist	Hepatoprotector herbal drug
				DUPILUMAB	antagonist	Biologic agents for uncontrolled atopic dermatitis, asthma, nasal polyposis
MED1	0.159	5.030	4.89e-07	BECOCALCIDIOL	NA	Investigational drug for psoriasis
PSMB9	0.041	-5.183	2.18e-07	CARFILZOMIB	inhibitor	Antineoplastic agent (oncohematology)
				BORTEZOMIB	inhibitor	Antineoplastic agent (oncohematology)
				IXAZOMIB CITRATE	inhibitor	Antineoplastic agent (oncohematology)
				MARIZOMIB	inhibitor	Investigational as antineoplastic

				OPROZOMIB	inhibitor	Investigational as antineoplastic
PSMD3	0.263	-5.869	4.37e-09	CARFILZOMIB	inhibitor	Antineoplastic agent (oncohematology)
				BORTEZOMIB	inhibitor	Antineoplastic agent (oncohematology)
				IXAZOMIB CITRATE	inhibitor	Antineoplastic agent (oncohematology)
				OPROZOMIB	inhibitor	Investigational as antineoplastic
RAD50	0.478	5.399	6.68e-08	IRINOTECAN	NA	Antineoplastic agents (solid cancer)
				AZD-7762	NA	Investigational as antineoplastic (checkpoint inhibitor)
				QUINPIROLE	NA	Experimental (psychoactive, neurologic disorders)
SLC22A5	0.189	8.831	1.03e-18	LEVOCARNITINE	NA	Amino acids derivatives (used in metabolic deficiency states)
SMAD3	1.000	6.206	5.41e-10	DEXAMETHASONE	NA	Corticosteroids (anti-inflammatory, immunosuppressive)
				GENISTEIN	NA	Experimental (antineoplastic, menopausal symptoms)
				HALOFUGINONE	NA	Experimental (malaria, cancer, and fibrosis-related and autoimmune diseases)
				LEUPRORELIN ACETATE	NA	Antineoplastic agent (hormone-sensitive tumors) and hormonal treatment
TAP2	0.0415	-5.490	4.01e-08	PRAMLINTIDE	agonist	Antidiabetic agent (amylin analog)
				CLOZAPINE	agonist	Antipsychotic
				ALCOHOL	agonist	Psychotropic substance
				CALCITONIN	agonist	Anti-parathyroid agent, bone antiresorptive agent
				MONOETHANOLAMINE	antagonist	Antivaricose therapy (local sclerosing agent)
				OLCEGEPANT	antagonist	Antimigraine

*Overall association score for asthma from the Open Targets Platform (PMID: 30462303)

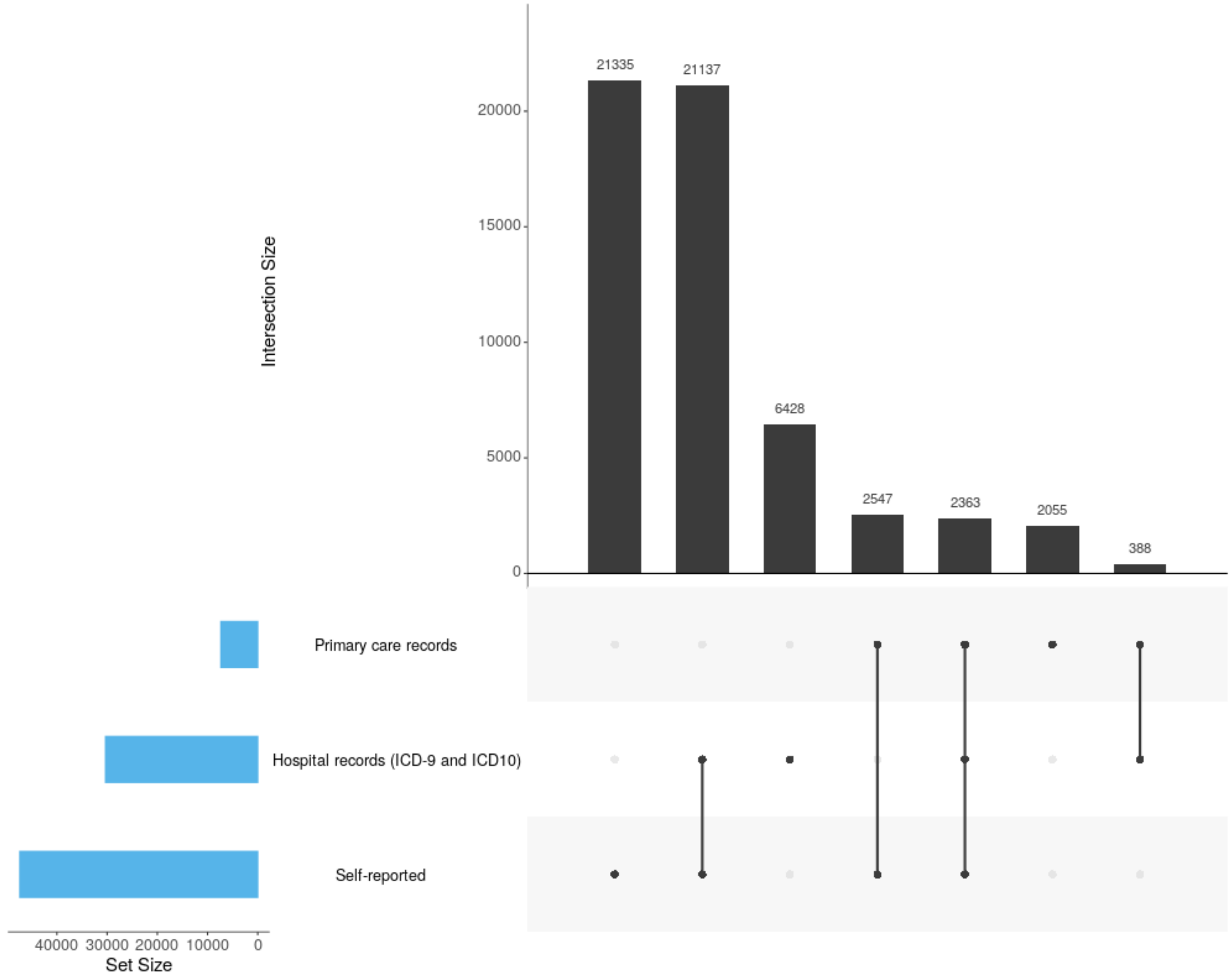

Supplementary Figure 1. UpSet plot showing the data sources to define asthma status in UK Biobank. A total of 56,167 asthma cases were identified. Affected individuals had one or more diagnosis of asthma based on self-reported questionnaires, hospital records (ICD-9 and ICD-10), and primary care records.

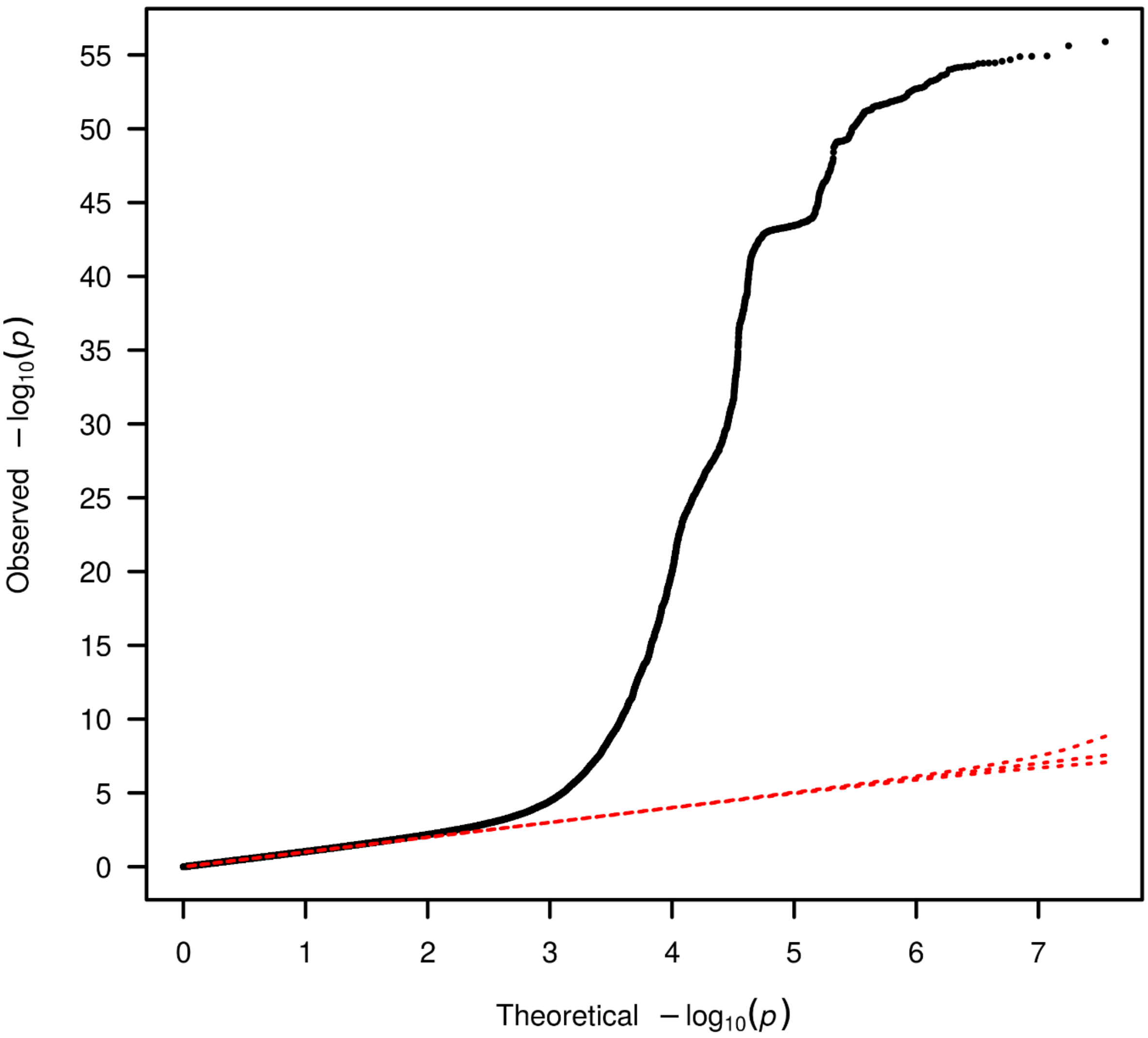

Supplementary Figure 2. Quantile-quantile plot of test statistics generated by the GWAS in UK Biobank including 56,167 asthma cases and 352,255 controls. Genomic inflation factor λ=1.029.

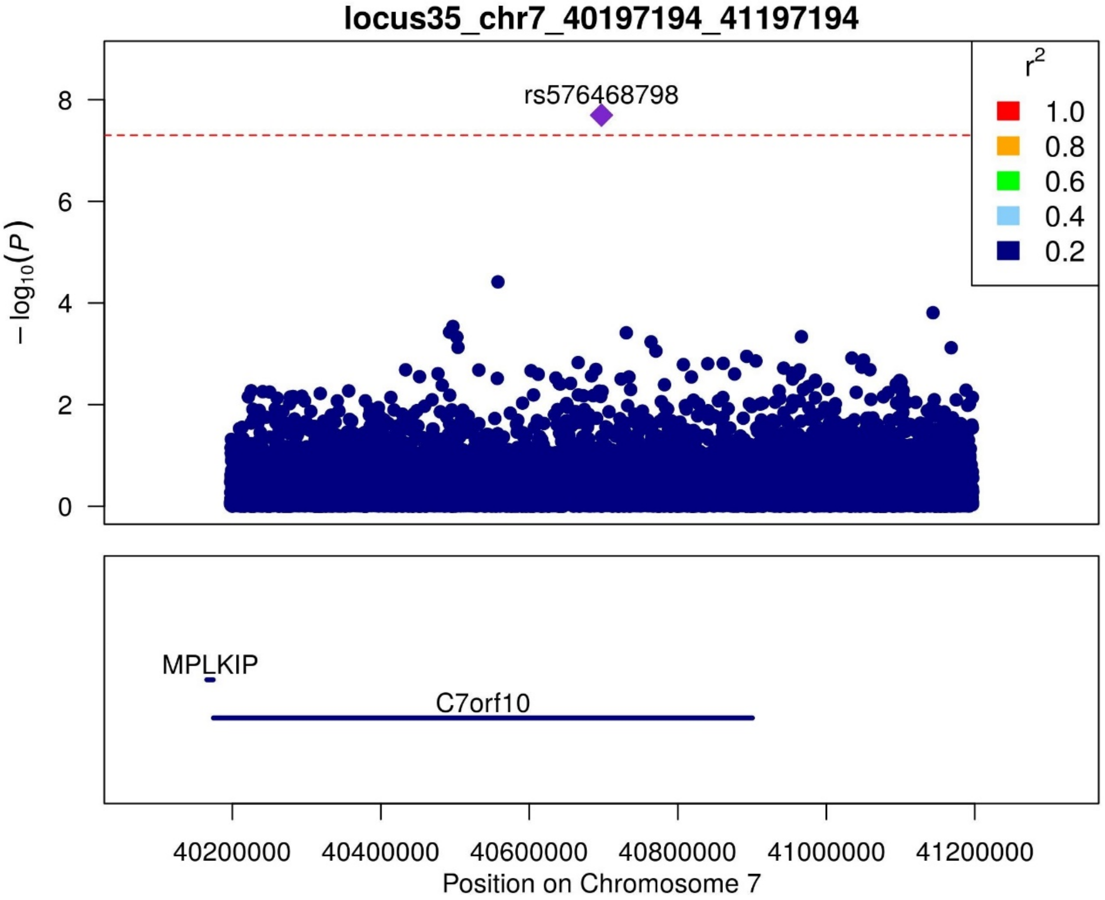

Supplementary Figure 3. Regional plot showing the 7p14 locus that passed the GWAS significance threshold, but only for one rare variant. The y axis shows the P value in -log10 scale for SNPs up- and downstream of the sentinel SNP (purple dot). The extent of linkage disequilibrium (LD; r^2 values) for all SNPs with the sentinel SNP is indicated by colors. The location of genes is shown at the bottom. SNPs are plotted based on their chromosomal position on build 37.

**Study design**	1) Main analysis	2) Lung disease	3) Smoking	4) Allergy
**Exclusion criteria**	-Samples that failed genotyping QC<br>-Non-white British ancestry	Study design 1 + cases and controls with COPD, emphysema, chronic bronchitis, interstitial lung disease or A1ATD	Study design 1 + cases and controls with a positive smoking history	Study design 1 + controls with atopy, including hay fever, allergic rhinitis, and eczema/atopic dermatitis
**Sample size**	Case n=56,167<br>Control n=352,255	Case n=47,391<br>Control n=340,033	Case n=21,097<br>Control n=136,586	Case n=56,167<br>Control n=268,142
**Effect size estimates and SE at the 72 asthma-associated loci**				

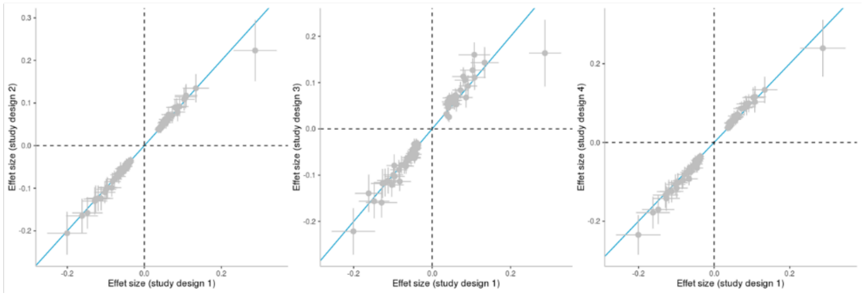

Supplementary Figure 4. Results of sensitivity analysis evaluating the potential confounder effects of other lung diseases, smoking and allergy. The three scatter plots compared the effect size estimates and SE of the main study design (x axis) with the three alternative study designs (y axis). The identity line is shown in blue. The single and most extreme outlier in the upper right corner of scatter plots consists of the sentinel variant on 1q21.3.

Lung TWAS

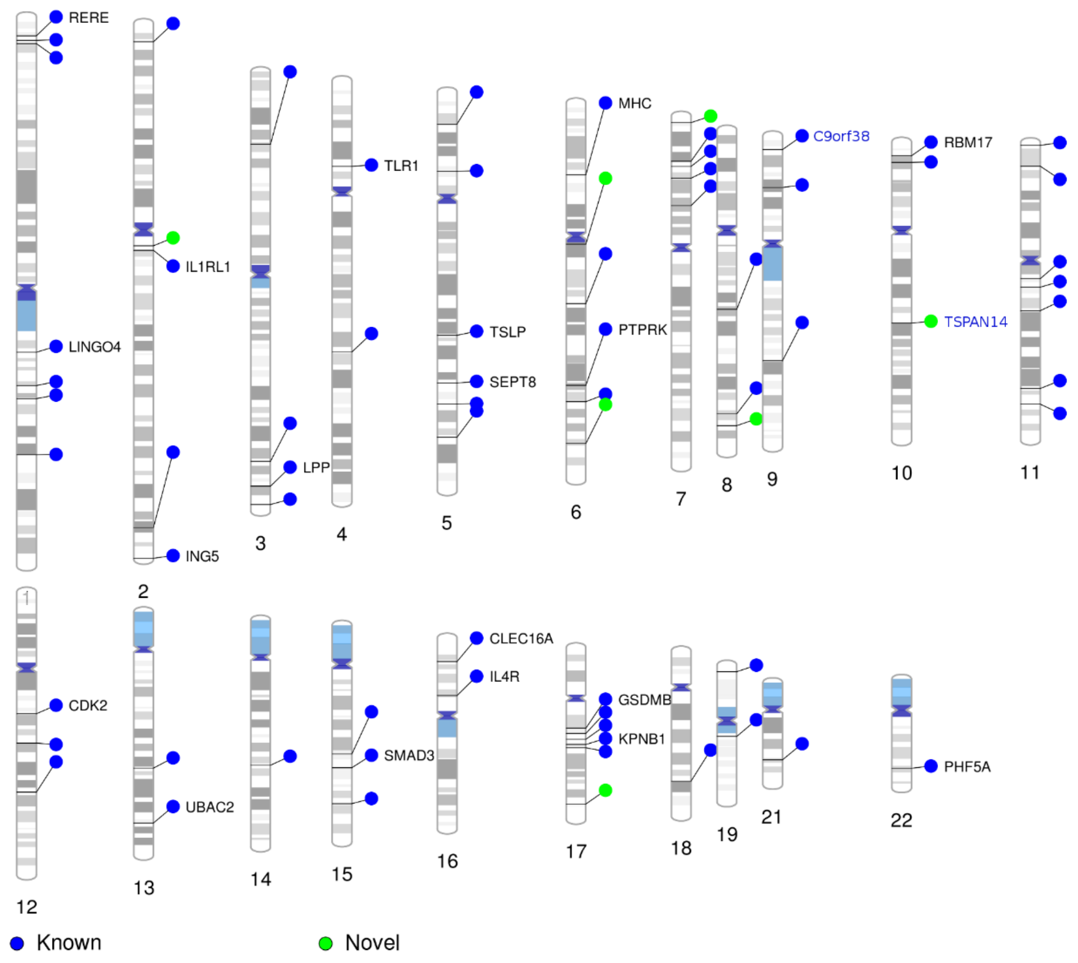

Supplementary Figure 5. The most significant lung TWAS genes identified per asthma-associated loci. Previously known and new asthma loci are illustrated in blue and green, respectively. Genes not reported in previous asthma GWAS are annotated in blue. Unlabeled blobs are asthma GWAS loci for which no significant lung TWAS gene was identified.

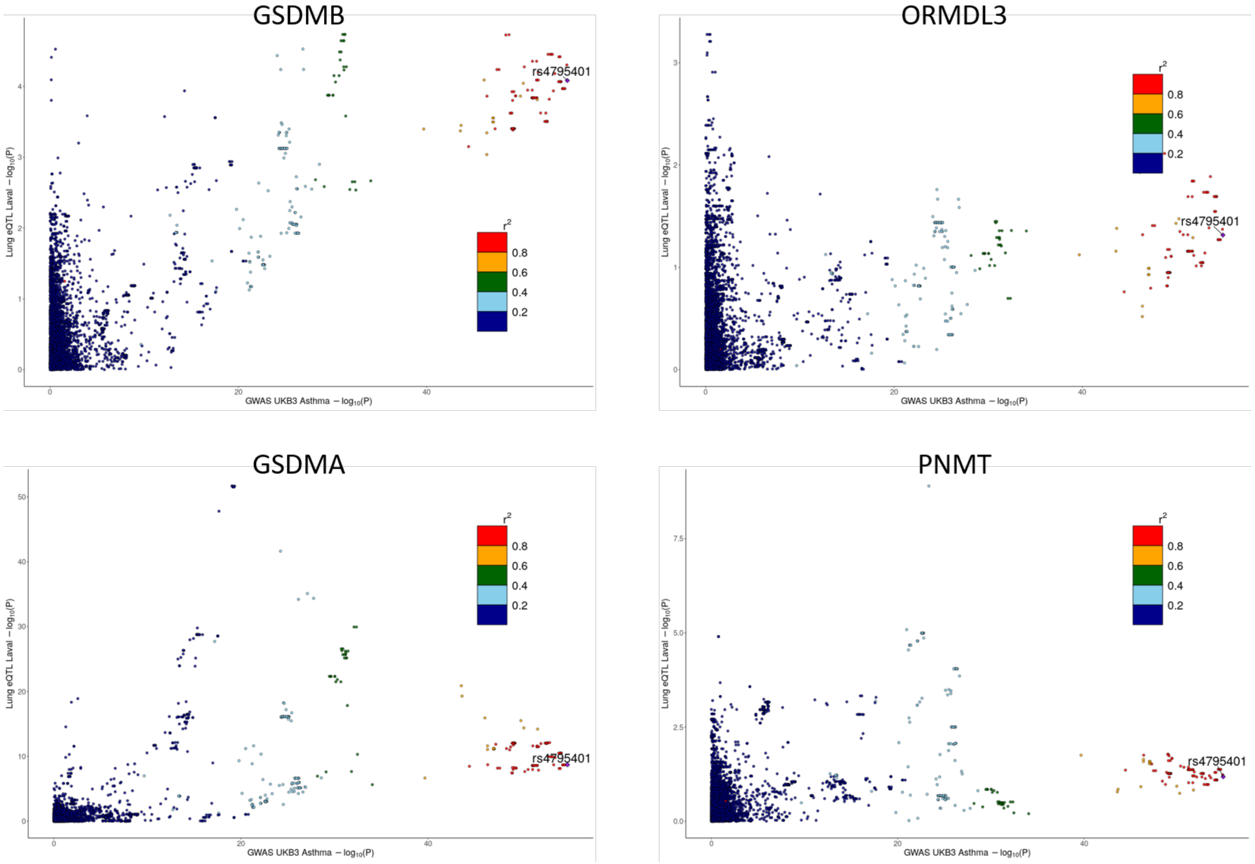

Supplementary Figure 6. LocusCompare plots for four significant TWAS genes on chromosome 17q12-q21. Association signals for SNPs within 50 Kb up and downstream of target genes are illustrated for *GSDMB*, *ORMDL3*, *GSDMA*, and *PNMT*.

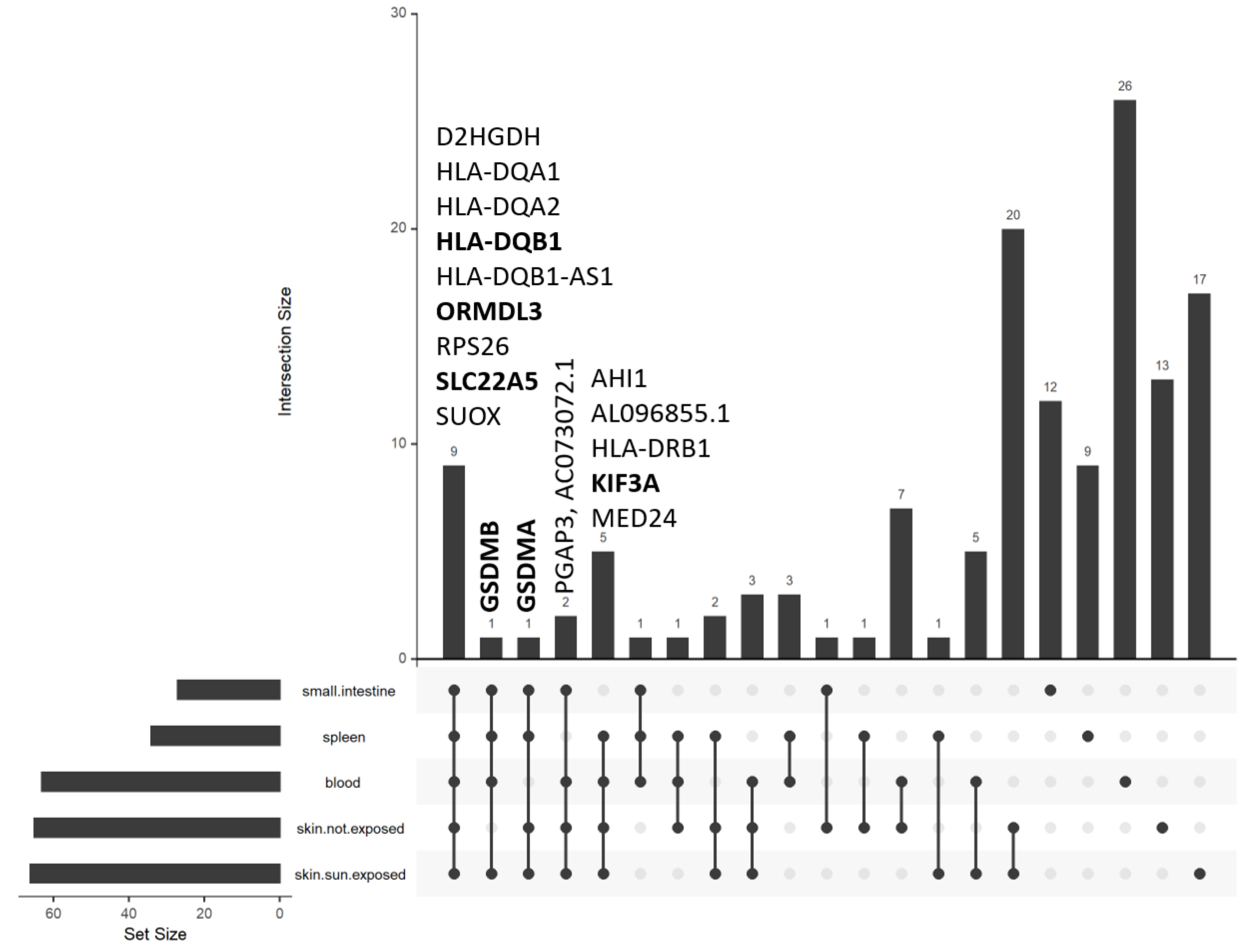

Supplementary Figure 7. Upset plot showing overlap of TWAS genes across GTEx tissues. TWAS genes found in four and five tissues are annotated. Lung TWAS genes are in bold.

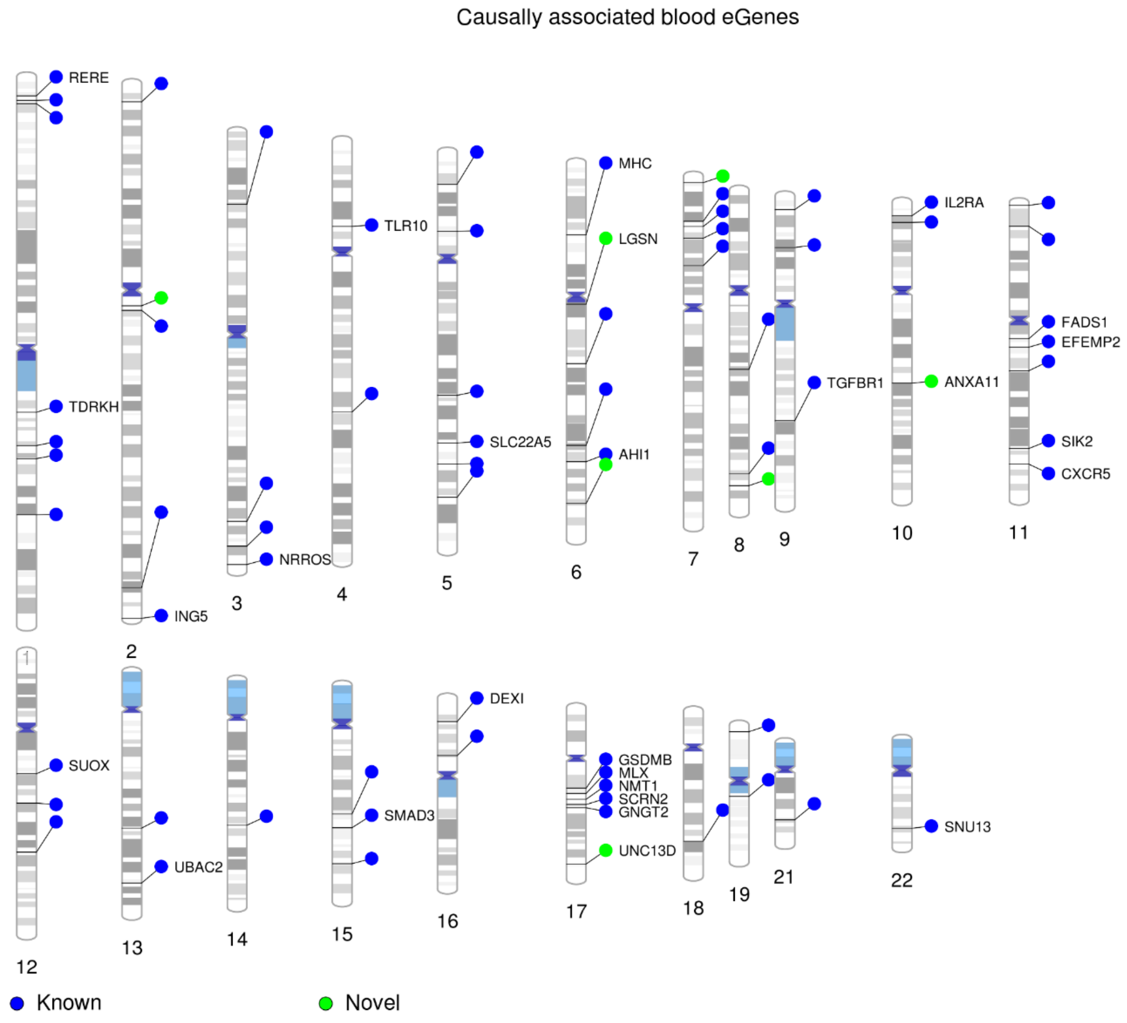

Supplementary Figure 8. The most significant causally associated blood eGene identified per asthma-associated loci. Previously known and new asthma loci are illustrated in blue and green, respectively. Unlabeled blobs are asthma GWAS loci for which no blood eGene was identified.

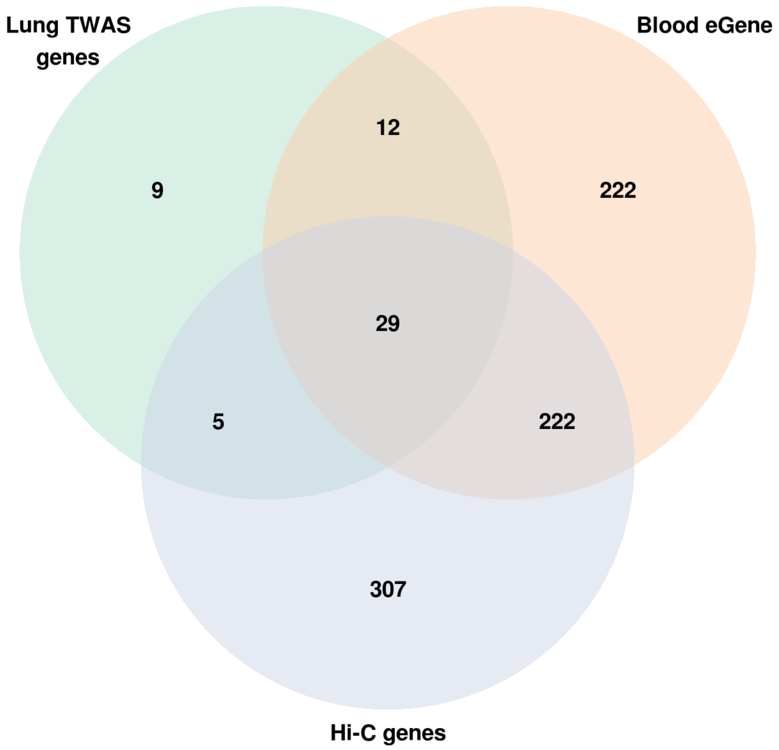

Supplementary Figure 9. Venn diagram showing the number of target genes that overlapped among 55 lung TWAS genes, 485 blood eGenes, and 563 chromatin contacts mapped genes.
