# Supplementary material for: Prioritization of candidate causal genes for asthma in susceptibility loci derived from UK Biobank
Source: Commun Biol. 2021 Jun 8;4:700. doi: 10.1038/s42003-021-02227-6 (PMC8187656; doi:10.1038/s42003-021-02227-6)
Supplement: Supplementary file 3 — Description of Supplementary Files [file 42003_2021_2227_MOESM3_ESM.pdf]

Description of Additional Supplementary Files

File name: Supplementary Data 1-20

Description:

Supplementary Data 1. The 73 physically defined asthma GWAS loci. The locus in red was excluded as indicated in the text.

Supplementary Data 2. The 116 independent genetic association signals of asthma in UK Biobank detected by conditional analysis. The locus in red was excluded as indicated in the text.

Supplementary Data 3. Assessment of novel signals at sixteen loci with more than one independent association signals.

Supplementary Data 4. Previous asthma GWAS variants that are not significant in this study.

Supplementary Data 5. Sensitivity analysis results for the sentinel variants in the four study designs.

Supplementary Data 6. Exonic variants associated with asthma (sorted by CADD scores).

Supplementary Data 7. Genome-wide significant lung tissue based TWAS genes of asthma.

Supplementary Data 8. Gene Ontology terms associated with lung TWAS genes associated with asthma (Human KEGG database).

Supplementary Data 9. Bayesian colocalization results for TWAS genes.

Supplementary Data 10. GARFIELD functional enrichment analyses of asthma-associated variants.

Supplementary Data 11. Significant blood eQTLs for SNPs associated with asthma.
Supplementary Data 12. Blood eGenes associated with asthma.

Supplementary Data 13. Gene Ontology terms associated with blood eGenes associated with asthma (Human KEGG database).

Supplementary Data 14. Chromatin contacts mapped genes in GM12878 and associated with asthma.

Supplementary Data 15. Two-sample Mendelian randomization on the 485 blood eGenes.

Supplementary Data 16. Identification of 50 asthma causally-associated eGenes in blood. In green are genes without heterogeneity and in orange are genes with heterogeneity but corrected by MR-PRESSO.

Supplementary Data 17. Druggability of the 806 target genes identified in this study. In green are 29 target genes that overlapped among lung TWAS genes, blood eGenes, and chromatin contact genes.

Supplementary Data 18. PheWAS for the 40 genes prioritized as therapeutic targets for asthma.

Supplementary Data 19. UK Biobank data fields and codes for excluding individuals in the three case-control subsets used in GWAS sensitivity analysis.

Supplementary Data 20. Demographic and clinical characteristics of the 1,038 subjects in the lung eQTL study.

File name: Supplementary Data 21

Description: Asthma TWAS in lung tissue. Results of S-PrediXcan integrating the UK Biobank asthma GWAS and the lung eQTL dataset (n=1,038).

File name: Supplementary Data 22

Description: Asthma TWAS in GTEx blood. Results of S-PrediXcan integrating the UK Biobank asthma GWAS and the blood eQTL dataset from GTEx (n=670).

File name: Supplementary Data 23

Description: Asthma TWAS in GTEx skin not sun exposed. Results of S-PrediXcan integrating the UK Biobank asthma GWAS and the skin not sun exposed eQTL dataset from GTEx (n=517).

File name: Supplementary Data 24

Description: Asthma TWAS in GTEx skin sun exposed. Results of S-PrediXcan integrating the UK Biobank asthma GWAS and the skin sun exposed eQTL dataset from GTEx (n=605).

File name: Supplementary Data 25

Description: Asthma TWAS in GTEx small intestine. Results of S-PrediXcan integrating the UK Biobank asthma GWAS and the small intestine eQTL dataset from GTEx (n=174).

File name: Supplementary Data 26

Description: Asthma TWAS in GTEx spleen. Results of S-PrediXcan integrating the UK Biobank asthma GWAS and the spleen eQTL dataset from GTEx (n=227).
