# Supplementary material for: Prioritization of candidate causal genes for asthma in susceptibility loci derived from UK Biobank
Source: Commun Biol. 2021 Jun 8;4:700. doi: 10.1038/s42003-021-02227-6 (PMC8187656; doi:10.1038/s42003-021-02227-6)
Supplement: Supplementary file 11 — Reporting Summary [file 42003_2021_2227_MOESM11_ESM.pdf]

nature research

Corresponding author(s): Yohan Bossé

Last updated by author(s): Apr 26, 2021

# Reporting Summary

Nature Research wishes to improve the reproducibility of the work that we publish. This form provides structure for consistency and transparency in reporting. For further information on Nature Research policies, see our Editorial Policies and the Editorial Policy Checklist.

## Statistics

For all statistical analyses, confirm that the following items are present in the figure legend, table legend, main text, or Methods section.

| n/a | Confirmed | |
|---|---|---|
| ☐ | ☒ | The exact sample size (*n*) for each experimental group/condition, given as a discrete number and unit of measurement |
| ☐ | ☒ | A statement on whether measurements were taken from distinct samples or whether the same sample was measured repeatedly |
| ☐ | ☒ | The statistical test(s) used AND whether they are one- or two-sided<br>*Only common tests should be described solely by name; describe more complex techniques in the Methods section.* |
| ☐ | ☒ | A description of all covariates tested |
| ☐ | ☒ | A description of any assumptions or corrections, such as tests of normality and adjustment for multiple comparisons |
| ☐ | ☒ | A full description of the statistical parameters including central tendency (e.g. means) or other basic estimates (e.g. regression coefficient) AND variation (e.g. standard deviation) or associated estimates of uncertainty (e.g. confidence intervals) |
| ☐ | ☒ | For null hypothesis testing, the test statistic (e.g. *F*, *t*, *r*) with confidence intervals, effect sizes, degrees of freedom and *P* value noted<br>*Give P values as exact values whenever suitable.* |
| ☐ | ☒ | For Bayesian analysis, information on the choice of priors and Markov chain Monte Carlo settings |
| ☐ | ☒ | For hierarchical and complex designs, identification of the appropriate level for tests and full reporting of outcomes |
| ☐ | ☒ | Estimates of effect sizes (e.g. Cohen's *d*, Pearson's *r*), indicating how they were calculated |

*Our web collection on statistics for biologists contains articles on many of the points above.*

## Software and code

Policy information about availability of computer code

| | |
|---|---|
| Data collection | UK Biobank<br>GTEx, version 8: https://www.gtexportal.org/home/<br>eQTLGen Consortium: https://www.eqtlgen.org/index.html<br>Hi-C data GM12878: https://www.ncbi.nlm.nih.gov/geo/query/acc.cgi?acc=GSE87112<br>DGIdb: www.dgidb.org<br>DrugBank: www.drugbank.ca<br>ChEMBL: www.ebi.ac.uk/chembl<br>PubChem: pubchem.ncbi.nlm.nih.gov<br>Open Targets Platform: https://www.targetvalidation.org/ |
| Data analysis | SAIGE, version 0.36.3.1: https://github.com/weizhouUMICH/SAIGE<br>LDSC, version 1.0.1: https://github.com/bulik/ldsc<br>GCTA, version 1.93.2beta: https://cnsgenomics.com/software/gcta/#COJO<br>S-PrediXcan: https://github.com/hakyimlab/MetaXcan<br>COLOC, version 3.2.1: https://cran.r-project.org/web/packages/coloc/index.html<br>LocusCompareR: version 1.0.0, https://github.com/boxiangliu/locuscomparer<br>HyPrColoc: https://github.com/jrs95/hyprcoloc<br>GARFIELD: version 2, https://www.ebi.ac.uk/birney-srv/GARFIELD/<br>FUMA: http://fuma.ctglab.nl<br>Enrichr: https://maayanlab.cloud/Enrichr/<br>MendelianRandomization: https://cran.r-project.org/web/packages/MendelianRandomization<br>MR-PRESSO: https://github.com/rondolab/MR-PRESSO |

LocusZoom: https://github.com/Geeketics/LocusZoms
UpSet plot: https://github.com/hms-dbmi/UpSetR
Chromosome ideogram: http://visualization.ritchielab.org/phenograms/document
GeneATLAS: http://geneatlas.roslin.ed.ac.uk
LDlink: https://ldlink.nci.nih.gov
dbNSFP: http://database.liulab.science/dbNSFP

For manuscripts utilizing custom algorithms or software that are central to the research but not yet described in published literature, software must be made available to editors and reviewers. We strongly encourage code deposition in a community repository (e.g. GitHub). See the Nature Research guidelines for submitting code & software for further information.

## Data

Policy information about availability of data

All manuscripts must include a data availability statement. This statement should provide the following information, where applicable:

- Accession codes, unique identifiers, or web links for publicly available datasets
- A list of figures that have associated raw data
- A description of any restrictions on data availability

The summary statistics for the asthma GWAS in UK Biobank (n=56,167 asthma cases and 352,255 controls) are available at The NHGRI-EBI Catalog of human genome-wide association studies: https://www.ebi.ac.uk/gwas/, study accession GCST90014325. The human lung tissue eQTL study is available in dbGaP under accession phs001745.v1.p1. The full summary statistics for the lung asthma TWAS (19,918 probe sets with significant gene expression prediction models) are available in the Supplementary Data 21. Summary statistics from the Trans-National Asthma Genetic Consortium were downloaded from the GWAS catalog: https://www.ebi.ac.uk/gwas/downloads/summary-statistics, study accession GCST006862.

# Field-specific reporting

Please select the one below that is the best fit for your research. If you are not sure, read the appropriate sections before making your selection.

☒ Life sciences ☐ Behavioural & social sciences ☐ Ecological, evolutionary & environmental sciences

For a reference copy of the document with all sections, see nature.com/documents/nr-reporting-summary-flat.pdf

# Life sciences study design

All studies must disclose on these points even when the disclosure is negative.

| | |
|---|---|
| Sample size | No sample-size calculation was performed. The largest possible sample size was used to perform the GWAS in UK biobank (n=56,167 asthma cases and 352,255 controls) as well as eQTL analysis in lung (n=1,038) and blood (n=31,684) tissues. |
| Data exclusions | The definition of asthma cases and controls in our study is based on the UK Biobank Outcome Adjudication Group. Samples with call rate <95%, outlier heterozygosity rate, sex mismatch, non-white British ancestry, samples with excess third-degree relatives (>10), or not used for relatedness calculation were excluded. |
| Replication | We checked for potential replication for the novel GWAS loci in summary statistics from the Trans-National Asthma Genetic Consortium (TAGC). Replication of the lung TWAS was performed in GTEx lung, with results shown in Table 3. |
| Randomization | For GWAS analysis, covariates included age, sex, and the first 20 ancestry-based principal components. GWAS sensitivity analysis was also performed to evaluate the potential confounding effect of other lung diseases, smoking, and allergy. Variables used to allocate asthma cases and controls are indicated in Supplementary Table 19. |
| Blinding | Blinding is not relevant for this study. |

# Reporting for specific materials, systems and methods

We require information from authors about some types of materials, experimental systems and methods used in many studies. Here, indicate whether each material, system or method listed is relevant to your study. If you are not sure if a list item applies to your research, read the appropriate section before selecting a response.

## Materials & experimental systems

| n/a | Involved in the study |
|---|---|
| ☒ | ☐ Antibodies |
| ☒ | ☐ Eukaryotic cell lines |
| ☒ | ☐ Palaeontology and archaeology |
| ☒ | ☐ Animals and other organisms |
| ☐ | ☒ Human research participants |
| ☒ | ☐ Clinical data |
| ☒ | ☐ Dual use research of concern |

## Methods

| n/a | Involved in the study |
|---|---|
| ☒ | ☐ ChIP-seq |
| ☒ | ☐ Flow cytometry |
| ☒ | ☐ MRI-based neuroimaging |

# Human research participants

Policy information about studies involving human research participants

| | |
|---|---|
| Population characteristics | UK Biobank: The present analyses were conducted under UK Biobank data application number 25205.<br>Lung eQTL: The lung eQTL dataset consists patients who underwent lung surgery at three academic sites, Laval University, University of British Columbia, and University of Groningen. |
| Recruitment | Lung eQTL: All lung specimens from Laval were obtained from patients undergoing lung cancer surgery and were harvested from a site distant from the tumor. At UBC, the majority of samples were from patients undergoing resection of small peripheral lung lesions. Additional samples were from autopsy and at the time of lung transplantation. At Groningen, the lung specimens were obtained at surgery from patients with various lung diseases, including patients undergoing therapeutic resection for lung tumors, harvested from a site distant from the tumor, and lung transplantation. |
| Ethics oversight | The study was approved by the Institut universitaire de cardiologie et de pneumologie de Québec – Université Laval (IUCPQ-UL) ethics committee. |

Note that full information on the approval of the study protocol must also be provided in the manuscript.
